# Effect of including procalcitonin and C-reactive protein in the Mortality in Emergency Department Sepsis risk prediction model

**DOI:** 10.1186/cc9693

**Published:** 2011-03-11

**Authors:** C Lee, J Liu, S Chen, S Chen

**Affiliations:** 1 National Taiwan University Hospital, Taipei, Taiwan; 2 Harvard School of Public Health, Boston, MA, USA; 3 Beth-Israel-Deaconess Hospital, Boston, MA, USA

## Introduction

The Mortality in Emergency Department Sepsis (MEDS) score has been gradually accepted as a reliable tool for bedside risk prediction of sepsis patients in the emergency department. Despite its clinical usefulness, the MEDS score did not take advantage of the prognostic information of biomarkers.

## Methods

We compared the clinical utility of MEDS score with and without CRP or PCT among participants in a prospective cohort of patients. All adult patients fulfilling the criteria for SIRS with a presumed infectious etiology were eligible for inclusion. Serum PCT and CRP were evaluated at admission. Initial severity was assessed with the MEDS score. Each patient was followed for at least 30 days for the 30-day survival. We built three extended models, including MEDS plus natural log PCT model (MEDS-LnPCT), MEDS plus natural log CRP model (MEDS-LnCRP), and MEDS plus natural log PCT and natural log CRP model (MEDS-LnPCT & LnCRP) for comparison. The values of CRP and PCT were transformed to natural log scale to normalize the distributions. We assessed whether adding CRP, PCT or both biomarkers to the MEDS model significantly reclassified patients into more appropriate risk categories. The reclassification was then evaluated by comparison of the observed incidence of events in the cells of the reclassification table with the predicted probability from the original MEDS model.

## Results

The 63 patients who died (10.6%) had significantly increased levels of PCT and CRP. Adjusting for MEDS predictors, either high levels of CRP or PCT was independently associated with 30-day mortality. We fitted PCT-incorporated (MEDS-PCT), CRP-incorporated (MEDS-CRP), and PCT & CRP incorporated (MEDS-PCT & CRP) models for comparison. The MEDS-PCT model was the favored model as it improved model fit and calibration as measured by the Net Reclassification Improvement (NRI) score (14.1%, *P *= 0.047). MEDS-CRP and MEDS-CRP & PCT models improved model fit (likelihood ratio test *P *= 0.03, 0.009, respectively) but did not improve calibration (NRI 5.4%, *P *= 0.204; 13.2%, *P *= 0.055). All three models did not improve model discrimination as measured by *c*-statistics.

## Conclusions

Adding PCT levels to the MEDS score reclassified patients into groups that better predict actual 30-day mortality. Inclusion of CRP or both biomarkers offers limited additional predictive value. Further validation studies are needed to corroborate these findings.

